# Thrombectomy for acute ischemic stroke patients with isolated distal internal carotid artery occlusion: a retrospective observational study

**DOI:** 10.1007/s00234-020-02550-5

**Published:** 2020-10-07

**Authors:** Jan W. Hoving, Manon Kappelhof, Mark Schembri, Bart J. Emmer, Olvert A. Berkhemer, Adrien E. D. Groot, Diederik W. J. Dippel, Wim H. van Zwam, Jonathan M. Coutinho, Henk A. Marquering, Charles B. L. M. Majoie, René van den Berg, for the MR CLEAN Registry Investigators

**Affiliations:** 1 Department of Radiology and Nuclear Medicine, Amsterdam UMC, University of Amsterdam, Amsterdam, The Netherlands; 2 Department of Biomedical Engineering and Physics, Amsterdam UMC, University of Amsterdam, Amsterdam, The Netherlands; 3 Department of Neurology, Amsterdam UMC, University of Amsterdam, Amsterdam, The Netherlands; 4 Department of Neurology, Erasmus MC, Rotterdam, The Netherlands; 5 Department of Radiology and Nuclear Medicine, Maastricht UMC, Maastricht, The Netherlands

**Keywords:** Stroke, Artery, Thrombectomy, CT angiography

## Abstract

**Purpose:**

Acute stroke patients presenting with a distal internal carotid artery occlusion and patent carotid terminus, allowing for collateral flow via the circle of Willis, may have a more favorable natural history. Therefore, benefit of endovascular treatment (EVT) is less evident. We performed an exploratory analysis of EVT results compared to conservative treatment in patients with ‘carotid-I’ occlusions.

**Methods:**

We report on EVT-treated and non-EVT-treated patients with carotid-I occlusions from the MR CLEAN Registry, MR CLEAN trial, and our comprehensive stroke center. CT-angiography was reviewed on primary collateral patency and choroid plexus enhancement. Perfusion deficits were assessed on CT-perfusion (CTP). Clot migration was assessed by comparing clot location on baseline CTA to its location on periprocedural digital subtraction angiography. Outcomes included 90-day functional independence (mRS 0–2), successful reperfusion and mortality.

**Results:**

We included 51 patients. Forty-one patients received EVT, ten patients did not. Intravenous thrombolysis was administered in 32 (78%) EVT-treated patients and 6 (60%) non-EVT-treated patients. CTP, available for 17 patients, showed hypoperfusion on cerebral blood flow maps in 13 (76%) patients. Successful reperfusion after EVT occurred in 23 (56%), and clot migration in 8 patients (20%). Functional independence was achieved in 54% (21/39) of EVT-treated and in 10% (1/10) of non-EVT-treated patients. Mortality was 26% (10/39) and 30% (3/10), respectively. Anterior choroidal artery patency and choroid plexus enhancement were positively associated with functional independence.

**Conclusion:**

In our population, data suggest improved outcomes after EVT in carotid-I occlusion patients and provide no arguments to withhold EVT in these patients.

**Electronic supplementary material:**

The online version of this article (10.1007/s00234-020-02550-5) contains supplementary material, which is available to authorized users.

## Introduction

Multiple clinical trials have proven the benefit of endovascular treatment (EVT) in patients with acute ischemic stroke caused by proximal occlusions of the anterior circulation, up to as much as 24 h after stroke onset [1–4]. For certain patients, however, the benefit of EVT remains unclear. Acute occlusion of the distal intracranial internal carotid artery (ICA) with an open ICA-terminus allows for substantial collateral flow to the middle cerebral artery (MCA) via the circle of Willis, mostly through the anterior communicating artery (ACOM). This occlusion pattern was previously described as “carotid-I occlusion” [5]. Due to the primary collateral flow, the natural history of these occlusions might be more favorable and the benefits of EVT may therefore be limited.

Several arguments can be made to either treat symptomatic patients with carotid-I occlusions with EVT or refrain from intervention and treat with intravenous thrombolysis (IVT) only, if eligible. Collateral flow in symptomatic patients with carotid-I occlusions is apparently not adequate with a risk for the development of watershed infarcts. In addition, proximal occlusion of the anterior choroidal artery branching off at the level of the distal ICA may cause internal capsule infarction, leading to worse outcome [6, 7]. Therefore, revascularization by IVT or EVT might be beneficial. However, early revascularization carries the risk of fragmentation or dislodgment of the clot into downstream territories leading to a more difficult disposition to reopen the territory involved [8]. Finally, symptoms may be caused by lacunar infarcts or distal occlusions not visible on CT-angiography (CTA), rather than the carotid-I occlusion itself. These considerations could be arguments against EVT.

As of yet, little data is available on treatment results in patients with carotid-I occlusions as they form a very small proportion of patients in stroke publications. Only 13% of all patients with carotid occlusions had the “carotid-I” configuration in the MERCI and Multi-MERCI trials [5]. Although patients with a causative occlusion of the ICA or M1 segment should receive EVT according to the current AHA/ASA Guidelines, [9] this recommendation is based on data of seven large EVT trials, where the carotid-I occlusion type was either not studied separately from other distal ICA-occlusions [10–12] or underrepresented [13]. It is very unlikely that the carotid-I occlusion pattern was prevalent enough to be adequately represented in the treatment effect estimated in these trials, leaving the optimal treatment approach for carotid-I occlusions unclear. Therefore, we aimed to perform an exploratory comparison of EVT versus conservative treatment in patients with carotid-I occlusions.

## Methods

### Patient inclusion

This study compares individual patient data from patients with a carotid-I occlusion who were treated with EVT, to carotid-I occlusion patients not treated with EVT. EVT-treated cases were included from the MR CLEAN Registry between March 2014 and November 2017, and from the MR CLEAN trial intervention arm between December 2010 and March 2014 [10]. Non-EVT-treated cases were included from the MR CLEAN trial control arm and from a consecutive cohort of carotid-I occlusion patients admitted to our center between 2012 and April 2018. All patients were treated with IVT if they were eligible [9].

The MR CLEAN Registry is an observational, prospective registry of all patients undergoing EVT for acute ischemic stroke in the Netherlands [14]. From the total population, we included patients with an anterior circulation occlusion, aged 18 years or older, who were treated in a MR CLEAN trial center within 6.5 h of symptom onset. We included cases with an occlusion of the distal intracranial ICA, without additional intracranial occlusions on CTA. In addition, patients were excluded if image quality was too poor for adequate assessment.

The MR CLEAN trial was a randomized clinical trial comparing usual care plus EVT to usual care alone [9]. Included patients were 18 years or older, had an acute ischemic stroke caused by a proximal occlusion in an anterior circulation artery, and presented within 6 h of symptom onset.

For the current study, we included patients with an intracranial carotid-I occlusion without additional intracranial occlusions on CTA. Patients with isolated extracranial carotid occlusions were not included.

The carotid-I occlusion patients from our local cohort were not treated with EVT and not included in the MR CLEAN trial control arm. EVT was withheld in these cases at the discretion of the interventionist: the risk of clot migration towards patent downstream territories was judged too large, or the potential benefit gained with EVT too small.

### Image analysis

All imaging was assessed by an independent core laboratory of (neuro)radiologists [10]. The current study used core lab data on early ischemic changes (Alberta Stroke Program Early CT Score; ASPECTS) on non-contrast CT (NCCT), CTA occlusion location (including possible intracranial occlusions distal to the carotid-I occlusion) and collateral score, reperfusion after EVT as assessed on digital subtraction angiography (DSA) images, and presence of symptomatic intracranial hemorrhage on follow-up NCCT.

For patients included in the current study, additional imaging assessments were performed by one experienced interventional neuroradiologist, who was blinded to all clinical information. Baseline NCCT and CTA images, as well as DSA images, were used to confirm presence of an intracranial carotid-I occlusion. We defined presence of a carotid-I occlusion as presence of contrast in the A1, carotid terminus, and M1 segments, with evidence of an intracranial thrombus in the distal ICA, from NCCT hyperdense artery sign, and/or CTA contrast cut-off, supported by DSA runs acquired before the first thrombectomy attempt.

Baseline CTA images were evaluated for patency of specific vessel segments relevant to the primary collateral circulation: the supra- and infraclinoid ICA, posterior cerebral artery (PCA) segment 1, ACOM, and left and right ACA segment 1, posterior communicating artery (PCOM), anterior choroidal artery (AChA), and ophthalmic artery. Absence of plexus enhancement of the choroid plexus in the temporal horn was assessed as a possible indirect sign for AChA occlusion. In addition, DSA images were compared with baseline CTA images to check for clot migration to distal territory. Furthermore, hypoperfusion, as measured by the cerebral blood flow (CBF) and cerebral blood volume (CBV), was assessed on baseline CT perfusion (CTP) for cases where CTP images were acquired.

### Outcome measures

Our primary outcome was 90-day functional independence (modified Rankin Scale score [mRS] 0–2). Secondary outcomes were ordinal 90-day mRS, mortality, successful reperfusion after EVT (extended thrombolysis in cerebral infarction [eTICI] score 2B-3), clot migration during EVT, delta-NIHSS (baseline-24 h NIHSS), and symptomatic intracranial hemorrhage (sICH), defined as a ≥ 4-point increase on the NIHSS and intracranial hemorrhage on follow-up NCCT.

### Statistical analysis

Because of low patient numbers, we performed descriptive, exploratory analyses only—no regression analyses or statistical testing could reliably be performed. Baseline, imaging, and outcome characteristics were compared between carotid-I occlusion cases treated with EVT and cases treated conservatively. In addition, we compared EVT-treated carotid-I occlusion cases with the overall MR CLEAN Registry population. Continuous variables were summarized as medians with interquartile ranges (IQR); categorical variables were noted as counts and percentages with 95% confidence intervals. All analyses were performed using R version 3.5.2 (R Foundation for Statistical Computing, Vienna, Austria).

## Results

A total of 51 patients met the inclusion criteria for this study: 41 underwent EVT (40/3352 [1%] of the MR CLEAN Registry patients; 1/500 [0.2%] of the MR CLEAN trial patients), ten did not (4 MR CLEAN trial patients, 6 local cohort) (Online Resource 1). Baseline patient characteristics compared with the overall Registry cohort are detailed in Table 1. Most patients were male (37/51; 73%). IVT was administered in 38 of 51 (75%) included patients. Four patients (8%) were treated with supportive care only. Patients did not receive IVT either due to presentation outside of the 4.5-h time window (5/13 patients [38%]) or due to contra-indications like recent surgery (1/13, 8%) for oral anticoagulation use (7/13, 54%). Baseline characteristics within each group divided by IVT-treatment are shown in Online Resource 2.

**Table 1:** Patient baseline characteristics

	EVT [*N* = 41]	Non-EVT [*N* = 10]	Registry overall [*N* = 3180]
Age (year), median (IQR)	62(55–76)	83(76–85)	72(61–81)
Male, *n*(%; 95%CI)	30(73; 5*8–84*)	7(70; *40–89*)	1654(52; *50–54*)
Baseline NIHSS, median (IQR) [known in]	15(8–21) [*N* = 40]	17(14–21)	16(11–19) [*N* = 3128]
Left hemisphere, *n*(%; 95%CI)	30(73; *58–84*)	5(50; *24–76*)	1686(53; *51–55*)
Hypertension, *n*(%; 95%CI) [known in]	12(30; *18–45*) [*N* = 40]	4(40; *17–69*)	1633(52; *51–54*) [*N* = 3118]
Atrial fibrillation, *n*(%; 95%CI) [known in]	6(14; *7–28*)	4(40; *17–69*)	756(24; *23–26*) [*N* = 3138]
Myocardial infarction, *n*(%; 95%CI) [known in]	8(20; *10–34*)	2(20; *6–0.51*)	441(14; *13–15*) [*N* = 3116]
Diabetes mellitus, *n*(%; 95%CI) [known in]	5(12; *5–26*)	3(30; *11–60*)	510(16; *15–17*) [*N* = 3156]
Pre-stroke mRS, median (IQR)	0(0–0)	1(0–3)	0(0–1)
Blood glucose level (mmol/l), median (IQR) [known in]	6.4(5.8–7.4) [*N* = 40]	6.9(5.9–9.2)	6.8(5.9–8.1) [*N* = 2813]
Smoking history, *n*(%; 95%CI) [known in]	12(29; *20–50*) [*N* = 36]	1(10; *2–40*)	677(28; *26–30*) [*N* = 2440]
Systolic blood pressure at baseline (mmHg), median (IQR) [known in]	151(126–170) [*N* = 40]	132(87–154)	150(132–165) [*N* = 3092]
Diastolic blood pressure at baseline (mmHg), median (IQR) [known in]	87(73–94) [*N* = 40]	80(65–90)	80(71–91) [*N* = 3084]
IVT administered, *n*(%; 95%CI) [known in]	32(78; *63–88*)	6(60; *31–83*)	2427(77; *75–78*) [*N* = 3169]
Onset-to-presentation time (min), median (IQR) [known in]	67(47–165) [*N* = 33]	70(58–101) [*N* = 9]	55(38–96) [*N* = 2572]
Door-to-needle time for IVT (min), median (IQR)	25(20–36) [*N* = 31]	34(24–56) [*N* = 6]	24(18–33) [*N* = 1919]
Onset-to-groin puncture time for EVT (min), median (IQR) [known in]	225(172–306)	NA	195(150–260) [*N* = 3264]

If the [known in] number is not shown, the variable was known in all patients. 95%CI in italics

*EVT* endovascular treatment, *IVT* intravenous thrombolysis, *IQR* interquartile range, *mRS* modified Rankin Scale, *N* number of patients, *NIHSS* National Institute of Health Stroke Scale, *SD* standard deviation

### Clinical outcomes

**Fig. 1:**
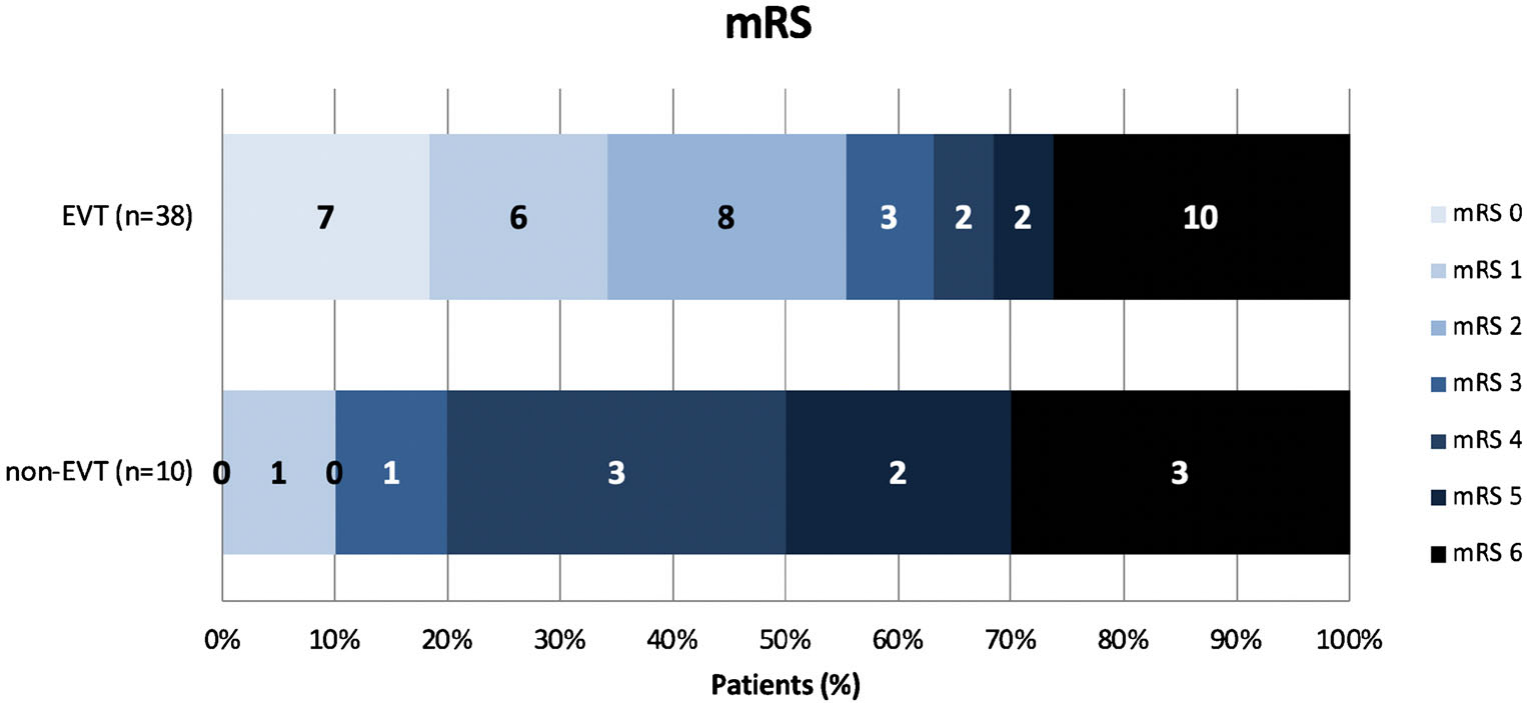
Comparative plot of the modified Rankin Scale (mRS) showing clinical outcomes for EVT- and Non EVT groups. 90-day mRS data was available for 38 patients in the EVT-treated group EVT=endovascular treatment, mRS=modified Rankin Scale score. Numbers in bars represent number of patients with corresponding mRS score

Table 2 shows clinical outcomes for EVT- versus non-EVT-treated patients. In our total population, data on 90-day functional outcomes were available for 49/51 (96%) patients. Twenty-two (43%) patients achieved functional independence compared with 38% (1200/3180) of patients in the overall MR CLEAN Registry cohort (Table 1). Overall, improved functional outcomes were observed in the EVT-treated group (Fig. 1). Functional independence after EVT was achieved in 14/23 (61%) of cases with successful reperfusion versus 7/18 (38%) of cases with unsuccessful reperfusion. In comparison, functional independence was achieved in 186/632 (29%) of Registry patients with carotid-T/L occlusions. Non-EVT-treated patients achieved functional independence in 1/10 (10%) of cases. Details on median delta-NIHSS, mortality at 90 days, and sICH are displayed in Table 2.

**Table 2:** Outcome data and imaging variables

	EVT [*N* = 41]	Non-EVT [*N* = 10]	Registry overall [*N* = 3180]
Functional independence (mRS 0–2) at 90 days, *n*(%; 95%CI) [known in]	21(54; *39–68*) [*N* = 39]	1(10; *2–40*)	1200(40; *39–42*) [*N* = 2968]
Ordinal mRS at 90 days, median (IQR) [known in]	2(1–6) [*N* = 39]	5(4–6)	3(2–6) [*N* = 2968]
Successful reperfusion after EVT, *n*(%; 95%CI) [known in]	23(56; *41–70*)	NA	1913(62; *60–63*) [*N* = 3096]
Mortality at 90 days, *n*(%; 95%CI) [known in]	10(26; *15–41*) [*N* = 39]	3(30; *11–60*)	863(29; *27–31*) [*N* = 2968]
Delta-NIHSS at 24 h, median (IQR) [known in]	6(0–11) [*N* = 38]	4(3–9) [*N* = 8]	4(0–9) [*N* = 2838]
sICH, *n*(%; 95%CI)	2(5; *1–16*)	1(10; *2–40*)	188 (6; *5–7*)
Patency of the infraclinoidal ICA, *n*(%; 95%CI)	5(12; *5–26*)	8(80; 4*9–94*)	NA
Patency of the supraclinoidal ICA, *n*(%; 95%CI)	13(32; *20–47*)	7(70; *40–89*)	NA
Patency of the PCOM, *n*(%; 95%CI)	14(34*; 22–49*)	6(60; *31–83*)	NA
Patency of the ACOM, *n*(%; 95%CI)	38(93; *81.97*)	10(100; *72–100*)	NA
Patency of the ACOM and PCOM (complete circle of Willis), *n*(%; 95%CI)	26(63; *48–76*)	4(40; *17–69*)	NA
Patency of the AChA, *n*(%; 95%CI)	18(44; *30–59*)	3(30; *11–60*)	NA
Patency of the ophthalmic artery, *n*(%; 95%CI)	14(34; *22–49*)	3(30; *11–60*)	NA
Plexus enhancement, *n*(%; 95%CI)	35(85; *72–93*)	5(50; *24–76*)	NA
Hypodensity on NCCT*, *n*(%; 95%CI)	10(24; *14–39*)	5(50; *24–76*)	NA
Hypodensity in internal capsule, *n*(%; 95%CI)	3(7; *3–19*)	4(40; *17–69*)	NA
ASPECTS at baseline, median (IQR) [known in]	10(9–10) [*N* = 39]	9(8–10) [*N* = 9]	NA
Collateral score, median (IQR) [known in]	3(2–3) [*N* = 37]	3(2.5–3) [*N* = 3]	NA
CBV reduction on CTP, *n*(%; 95%CI) [known in]	5(50; *24–76*) [*N* = 10]	5(71; *36–92*) [*N* = 7]	NA
CBF reduction on CTP, *n*(%; 95%CI) [known in]	6(60; *31–83*) [*N* = 10]	7(100; *65–100*) [*N* = 7]	NA
CBF/CBV mismatch on CTP, *n*(%; 95%CI) [known in]	5(50; *24–76*) [*N* = 10]	3(43; *16–75*) [*N* = 7]	NA

If the [known in] number is not shown, the variable was known in all patients. 95%CI in italics

*AchA* anterior choroidal artery, *ACOM* anterior communicating artery, *CBF* cerebral blood flow, *CBV* cerebral blood volume, *CTP* CT perfusion, *Delta-NIHSS* NIHSS at baseline minus NIHSS at 24 h after stroke, *EVT* endovascular treatment, *ICA* internal carotid artery, *IQR* interquartile range, *IVT* intravenous thrombolysis, *M1* first segment of middle cerebral artery, *min* minutes, *mRS* modified Rankin Scale score, *N* number of patients, *NA* not applicable/available, *NIHSS* National Institute of Health Stroke Scale, *sICH* symptomatic intracranial hemorrhage

*Hypodensity on NCCT is defined as ASPECTS ≤ 9

### Imaging findings

A detailed overview of all imaging variables, subcategorized for EVT- versus non-EVT-treated patients, is shown in Table 2. In our total population, initial (pretreatment)non-contrast CT scan showed hypodensity in the ipsilateral hemisphere in 15/51 (29%) patients. Enhancement of the choroid plexus was found in 40/51 (78%) patients. Higher rates of functional independence were seen in EVT-treated patients with patency of the anterior choroidal artery (63 vs. 50%), patency of the ophthalmic artery (67 vs. 50%), a complete circle of Willis (50 vs. 42%), and choroid plexus enhancement (56 vs. 50%).

CTP was available in 17/51 patients (33%). Hypoperfusion, as measured with CBF, was seen in 13 patients (Table 2). A core infarct, as measured with CBV, was visible in 10 patients. In all cases, perfusion deficit on CTP involved the entire hemisphere or multiple flow areas. Of the 10 patients with a core infarct visible on CTP, five patients showed CBF/CBV mismatch indicating presence of a penumbra.

Procedural details on EVT treated patients are given in Online Resource 3 in eight (20%) EVT-treated patients, clot migration to the proximal MCA, ACA, or PCA branches had occurred during the procedure, with failure to remove the dislodged clot. In five of these patients, the distal embolus was located in the MCA territory—none of these patients were functionally independent at 3 months post-stroke. In the other three cases, clot fragmentation and migration occurred into the ACA and/or PCA territory. Three-month outcome was mRS 1 for one of these patients and mRS 4 in the other two.

Occlusion location on the first run of DSA differed from the CTA occlusion location for two cases in our study: both clots had migrated from the carotid top to the proximal M1 segment and were successfully removed during EVT.

### Illustrative case

**Fig. 2:**
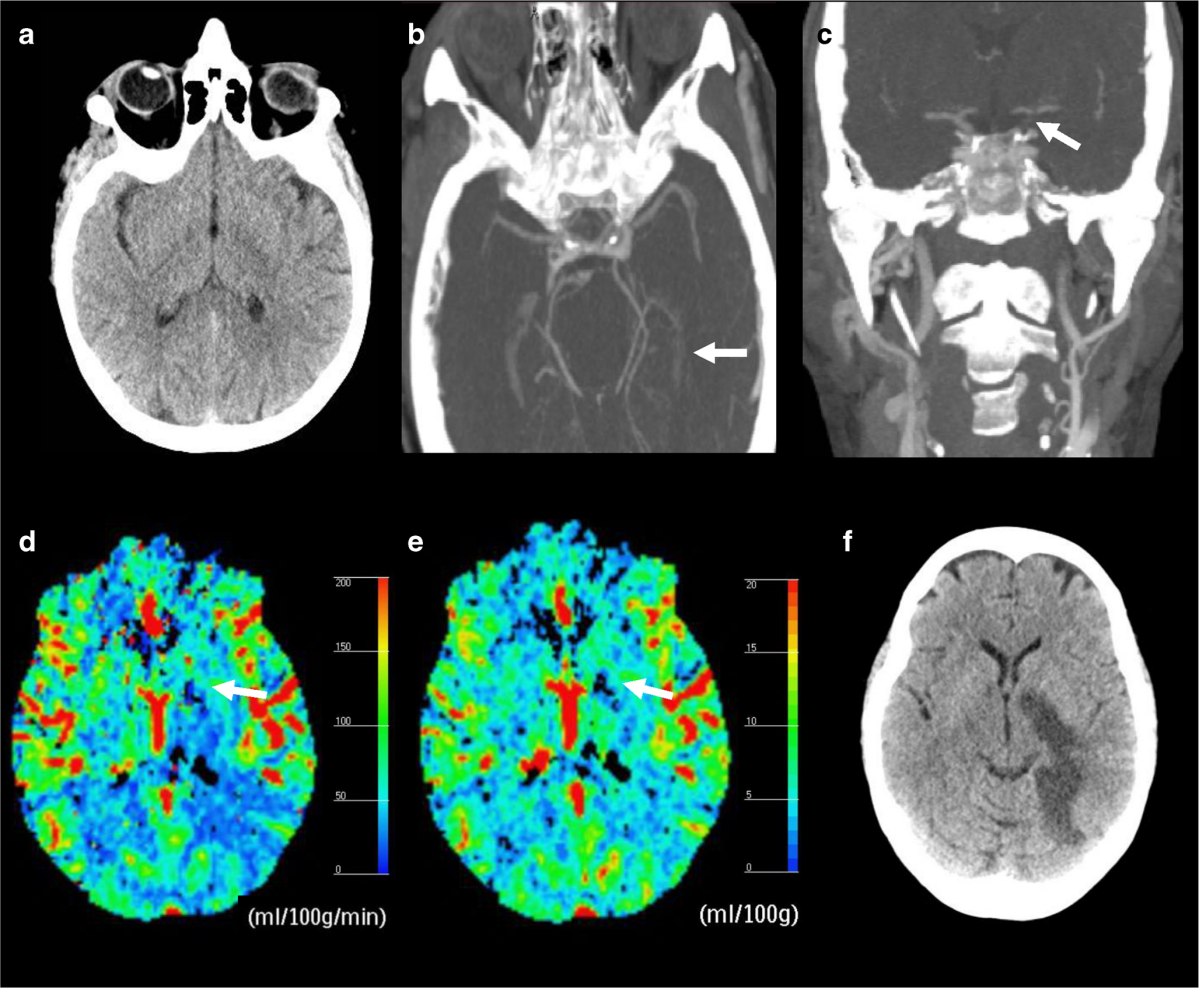
Illustrative case a: Baseline non-contrast CT showing subtle region of hypo-attenuation in the left medial temporal lobe and posterior limb of the internal capsule. b: Near-absent enhancement of left choroid plexus (arrow) and patent PCOM and PCA. c: Coronal CT-angiography MIP shows distal left carotid-I occlusion (arrow) with patent middle and anterior cerebral arteries. d: Cerebral blood flow map of CT perfusion subtly showing less flow in the left genu/posterior limb of internal capsule (arrow), lentiform nucleus and temporal lobe area. Scale presented on right side of image. e: Cerebral blood volume map showing decreased volume in genu/posterior limb of internal capsule (arrow). f: 5-day follow-up non-contrast CT showing large infarct in the left internal capsule and temporal lobe

A 63-year-old patient was admitted 3 h after last seen well with right-sided paralysis and speech difficulties. Medical history was unremarkable for cardiovascular events and risk factors. Initial neurological examination showed an NIHSS of 20. A gaze paresis with horizontal eye deviation towards the left side was present together with a right-sided facial palsy without upper face involvement, a complete paralysis of the right arm and paresis of the right leg, and a right-sided hemianopia. NCCT showed a subtle area of hypo-attenuation in the left medial temporal lobe and posterior limb of the internal capsule (Fig. 2a). Baseline ASPECTS was 7. CTA at presentation showed a distal ICA occlusion but normal patency of the MCA, ACA, and PCA (Fig. 2b). Moreover, CTA showed only faint enhancement of the left choroid plexus and patent left PCOM and PCA (Fig. 2c). CTP data were post-processed using Philips Extended Brilliance Workspace v3.5 (Philips Healthcare, Best, Netherlands). CBF and CBV maps showed impaired perfusion on CTP in the left genu/posterior limb of the internal capsule and lentiform nucleus. (Fig. 2d, e). The patient received IVT as per protocol but lacked clinical improvement. No EVT was performed as the patient was allocated to the control arm of a randomized trial. The patient had no adverse events during admission and showed mild clinical improvement (NIHSS at discharge 17). Follow-up CTA showed no recanalization of the primary occlusion. Follow-up NCCT showed a large infarct of the medial part of the temporal lobe and posterior limb of the internal capsule (Fig. 2f). The extent of the infarct can be attributed to the occlusion of the anterior choroidal artery, which also supplies a large cortical territory. At 3 months follow-up, the patient was unable to walk unassisted, required help with daily activities, and was unable to live independently, corresponding to mRS 3.

## Discussion

This explorative study examining the results of EVT in patients with ischemic stroke due to isolated distal internal carotid artery occlusion (“carotid-I occlusion”) suggests increased rates of functional independence and decreased mortality rates after EVT compared with conservative treatment, including IVT if patients are eligible.

Severe neurological deficits in cases of carotid-I occlusion with crossflow circulation through the circle of Willis and a patent ACA and MCA can be explained by hemodynamic dysfunction of the circle of Willis, leading to watershed infarcts, or by infarction of the anterior choroidal artery territory. Alternatively, neurological deficit in carotid-I occlusion cases may be, at least partially, explained by small cortical thrombi in the distal vasculature. However, CTP in our patients showed perfusion deficits in the entire hemisphere or multiple flow territories, making the observed loss of function more likely attributable to a proximal occlusion. Moreover, rates of functional independence in patients without successful reperfusion were considerably lower than those in patients with successful reperfusion, indicating benefit of successful removal of carotid-I thrombi.

Although EVT is a well-proven indication for large vessel occlusions including distal ICA and MCA occlusions according to most recent guidelines [9], carotid-I occlusion should be considered as a different entity. The risk of treatment is much higher in patients with a patent intracranial circulation due to the risk of distal clot migration than in patients with a complete flow arrest. When clot migration occurred during the procedure, patients in our sample showed poor outcomes. Clot migration rates may decrease over time with evolving interventional techniques, potentially resulting in further improved outcomes after EVT in future patients with carotid-I occlusions. In addition, the exact stroke target can be either restoration of the diminished hemispheric perfusion or reopening of the occluded anterior choroidal territory, occlusion of which might lead to more definitive, irreversible neurological deficits.

Current AHA/ASA guidelines do recommend EVT for patients with intracranial ICA occlusions [9]. However, these recommendations are based on the large EVT trials. Of those, the MR CLEAN (*n* = 500), ESCAPE (*n* = 316), and SWIFT PRIME (*n* = 196) trials only specified carotid-T, carotid-L/T, and carotid-T occlusions in their inclusion criteria, respectively, and did not describe carotid-I occlusion cases separately [10–12]. In the REVASCAT trial (*n* = 206), carotid-I occlusions were mentioned as a separate group, resulting in one single patient with this occlusion type included in the control group. Since the intracranial collateral circulation is sustained in carotid-I occlusions through the circle of Willis, a more favorable natural course and smaller EVT benefit may be expected. Hence, the treatment effect of EVT in carotid-I occlusions cannot be concluded from the large EVT trial results.

Previous studies on carotid-I occlusions report on a limited number of patients. First, Liebeskind et al. described nine carotid-I occlusion cases treated with the Merci Retrieval System [5]. They reported a lower rate of functional independence at 90 days of 38%, possibly due to the use of an older generation thrombectomy device. Second, a recent study by Kim et al. assessed treatment results in a Korean population with ICA occlusion and patent collateral MCA flow, with or without additional intracranial occlusions [15]. They included eight patients with carotid-I occlusions treated with medical treatment only and 27 treated with EVT, but unfortunately, no outcome data on the carotid-I subgroup was presented. Third, a recent study in a Japanese population found poorer outcomes in carotid-I occlusion patients with clot migration before or during EVT, corresponding to our observations on clot migration [16].

Good collateral flow through the ACOM or PCOM can effectively sustain the viability of the brain parenchyma without causing any kind of neurological deficit in carotid-I occlusions, as is observed in balloon occlusion testing before permanent occlusion of the ICA to treat carotid aneurysms [17]. Without prior balloon occlusion testing, approximately 70% of patients will remain asymptomatic [17]. Hence, approximately 70% of the general population can be expected to not show any clinical symptoms in case of an acute distal carotid-I occlusion. If a patient presents with an acute neurological deficit and a carotid-I occlusion, this implies hypoperfusion. Hypoperfusion in this setting can be caused by failure of the collateral system, with or without concomitant cortical infarction due to distal thrombi. Patients with more severe strokes at baseline can hence be expected to have more circle of Willis anomalies, as was confirmed in this study’s imaging readings. Even in situations where only minor deficits are present, prolonged hypoperfusion may finally lead to progressive neurological deficits if treatment is withheld [18–22]. Also, carotid-I occlusions secondary to atherosclerotic plaque may be more challenging technically and lead to less favorable outcome rates compared with carotid-I occlusions of embolic origin [23].

Theoretically, CTP imaging could depict the extent of the territory at risk, pointing to the necessity of urgent revascularization of the distal ICA, even if all intracranial arteries are patent on CTA. CTP could also show perfusion deficits in the internal capsule and other AChA-related territories. Unfortunately, CTP imaging was only available in one third of EVT-treated patients. Hypoperfusion on CTP, as measured by both CBF and CBV, could give support to start EVT, although the results in our patient sample should be interpreted cautiously given that in our population only 6/17EVT-treated patients showed hypoperfusion on CTP.

If no CTP imaging is available, CTA can show lack of enhancement of the choroid plexus, pointing to a perfusion problem in the AChA territory [22]. Presence of a clot at the distal ICA can occlude the origin of the AChA. Emboli or insufficient collateral supply can subsequently cause infarction in the posterior limb of the internal capsule and the basal ganglia parts supplied by the AChA. Ischemia in this territory can lead to severe neurological deficits such as hemiplegia (pyramidal tract), hemianesthesia (thalamus and thalamo-cortical fibers), and hemianopia (optic tract, lateral geniculate body, or geniculo-calcarine tract).

Usually, AChA origin occlusions do not lead to clinical sequelae because of the connections in the choroid fissure with the choroidal branches from the PCA [24]. The tolerance of AChA occlusion correlates with absence of AChA infarction or restriction of ischemia to the medial temporal artery area only [25]. Especially in case of anatomical variations of the AChA and PCA territories (illustrative case), an AChA origin occlusion could lead to enlargement of the ischemic area including involvement of the medial part of the temporal, occipital, and parietal lobes, resulting in a more profound neurological deficit. The illustrative case represents the Takahashi Type 2 variation: an anomalous temporal artery supplying the infero-medial part of the temporal lobe [26].

Several limitations of the current study should be noted. First, although to our knowledge this study describes the largest cohort of patients with carotid-I occlusions published so far, the low number of patients may result in accidental findings. Second, selection bias may have occurred in the non-EVT-treated group due to the retrospective, non-randomized nature of our data: the EVT-treated group was probably selected based on a more favorable profile in their baseline characteristics. We observed a lower age, lower pre-stroke mRS, and lower baseline NIHSS in EVT-treated patients. This could also explain the observation that patients with unsuccessful reperfusion (eTICI 0–1) showed better functional outcome than patients in the non-EVT group. Third, occlusion location was scored on CTA in this study, whereas DSA can more accurately show occlusion pattern and patency of small branches. However, since the decision to treat endovascularly is made based on CTA in daily practice, we believe that this approach is more realistic and clinically applicable. Fourth, we have no data on recanalization status of non-EVT-treated patients, nor post-treatment parameters like blood pressure fluctuation, which was reported to be associated with penumbral tissue loss in non-recanalized patients [27]. Fifth, we excluded patients in whom isolated extracranial ICA occlusions were the cause of stroke. This was done to homogenize our study sample and focus on intracranial carotid-I occlusions, specifically. The patient group with acute ischemic stroke and isolated extracranial ICA occlusions poses a currently relevant clinical topic for further study. Finally, our findings are based on observational data. Ideally, randomized data without selection bias would be necessary to confirm the effect of EVT in patients with carotid-I occlusion and acute neurological deficit, though these data will be difficult to acquire due to the low prevalence of carotid-I occlusions. Our observations can serve as hypothesis generating for future studies on this topic.

## Conclusion

In our population, we found overall improved outcomes after EVT in patients with a carotid-I occlusion and acute clinical symptoms, though the patients in our study sample with unsuccessful reperfusion or unsuccessfully retrieved distal emboli after EVT showed poor clinical outcomes. Our data suggest that EVT should be considered in carotid-I occlusion patients, though additional, ideally randomized, research would be necessary to confirm these findings.

## Electronic Supplementary Material

ESM 1 (PDF 177 kb).

ESM 2 (PDF 175 kb).

ESM 3 (PDF 152 kb).
