# Supplementary material for: Thrombectomy for acute ischemic stroke patients with isolated distal internal carotid artery occlusion: a retrospective observational study
Source: Neuroradiology. 2020 Oct 7;63(5):777–86. doi: 10.1007/s00234-020-02550-5 (PMC8041676; doi:10.1007/s00234-020-02550-5)
Supplement: Supplementary file 1 — (PDF 177 kb). [file 234_2020_2550_MOESM1_ESM.pdf]

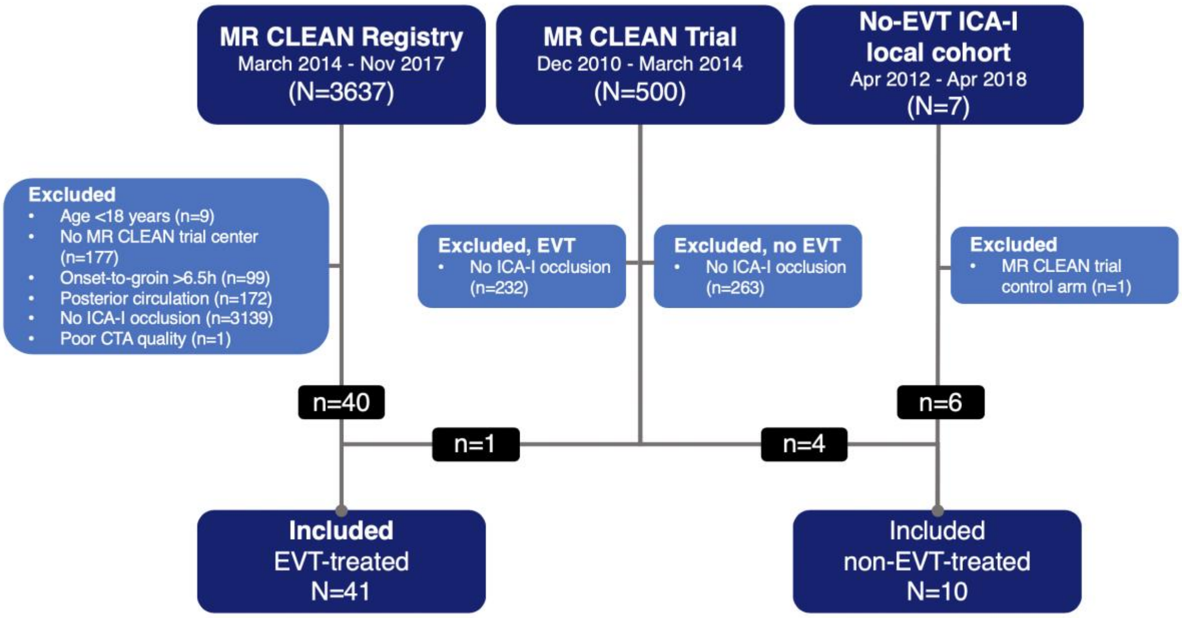

Online Resource 1 – Patient inclusion flow chart from the MR CLEAN Registry, MR CLEAN trial, and local non-EVT-treated cohort. *CTA=CT angiography, EVT=endovascular treatment, ICA=internal carotid artery, MR CLEAN=Multicenter Randomized Clinical Trial of Endovascular treatment for Acute ischemic stroke in the Netherlands.*
