# Supplementary material for: Thrombectomy for acute ischemic stroke patients with isolated distal internal carotid artery occlusion: a retrospective observational study
Source: Neuroradiology. 2020 Oct 7;63(5):777–86. doi: 10.1007/s00234-020-02550-5 (PMC8041676; doi:10.1007/s00234-020-02550-5)
Supplement: Supplementary file 2 — (PDF 175 kb). [file 234_2020_2550_MOESM2_ESM.pdf]

	EVT only [N=9]	IVT+EVT [N=32]	IVT only [N=6]	Supportive care only [N=4]
**Mean age** – yr (SD)	63 (17)	63 (15)	84 (11)	82 (9)
**Sex** – n(%) male	4 (78)	23 (72)	4 (67)	3 (75)
**Baseline NIHSS** – median (IQR) [known in]	20 (11-22) [N=8]	14 (8-21)	17 (13-20)	19 (14-23)
**Left hemisphere** – n(%)	6 (67)	24 (75)	3 (50)	2 (50)
**Hypertension** – n(%)	4 (44)	8 (25)	3 (50)	1 (25)
**Atrial fibrillation** – n(%)	3 (33)	3 (9)	2 (33)	2 (50)
**Myocardial infarction** – n(%)	3 (33)	5 (16)	2 (33)	0 (0)
**Diabetes mellitus** – n(%)	1 (11)	4 (13)	1 (67)	2 (67)
**Blood glucose level (mmol/l)** – median (IQR) [known in]	6.4 (5.9-6.8) [N=8]	6.4 (5.7-7.6) [N=29]	6.5 (5.9-7.6)	9.5 (6.0-10.1)
**Smoking history** – n(%)	3 (33)	9 (28)	0 (0)	1 (25)
**Systolic blood pressure at baseline (mmHg)** – median (IQR) [known in]	144 (140-175)	155 (126-170) [N=31]	143 (87-154)	126 (87-168)
**Diastolic blood pressure at baseline (mmHg)** – median (IQR)	90 (77-98)	84 (72-94) [N=31]	75 (49-86)	85 (73-116)
**Onset-to-presentation at EVT-center time (min)** – median (IQR) [known in]	126 (56-215) [N=8]	62 (47-105) [N=25]	64 (43-94)	101 (75-101) [N=3]
**Door-to-needle time (min)** – median (IQR) [known in]	NA	25 (20-36) [N=27]	34 (24-56)	NA
**Functional independence (mRS 0-2) at 90 days** - n(%) [known in]	2 (22) [N=8]	19 (59) [N=30]	1 (17)	0 (0)
**Successful reperfusion after EVT** – n(%)	5 (56)	18 (56)	NA	NA
**Mortality at 90 days** – n(%) [known in]	3 (33) [N=8]	7 (22) [N=31]	2 (33)	1 (25)
**Delta-NIHSS at 90 days** – median (IQR) [known in]	2 (1-11) [N=8]	7 (0-11) [N=30]	3 (3-7)	0 (0-6)
**sICH** – n(%)	0 (0)	2 (6)	1 (17)	0 (0)
**Patency of the infraclinoidal ICA** – n(%)	3 (33)	2 (6)	4 (67)	4 (100)
**Patency of the supraclinoidal ICA** – n(%)	2 (22)	11 (34)	4 (67)	3 (75)
**Patency of the PCOM** – n(%)	5 (56)	9 (28)	4 (67)	2 (50)
**Patency of the AChA** – n(%)	6 (67)	12 (38)	1 (17)	2 (50)
**Patency of the ACOM** – n(%)	8 (89)	30 (94)	6 (100)	4 (100)
**Patency of the ophthalmic artery** – n(%)	0 (0)	14 (44)	1 (17)	2 (50)
**Plexus enhancement** – n(%)	8 (89)	27 (84)	1 (17)	4 (100)

**Hypodensity on NCCT** – n(%)	1 (11)	9 (28)	2 (33)	3 (75)
**ASPECTS at baseline** – median (IQR) [known in]	9 (8-10)	10 (9-10)	10 (8-10)	8 (8-9)
**CBV reduction on CTP** – n(%) [known in]	0 (0) [N=2]	5 (63) [N=8]	3 (60) [N=5]	2 (50) [N=2]
**CBF reduction on CTP** – n(%) [known in]	0 (0) [N=2]	6 (75) [N=8]	5 (100) [N=5]	2 (50) [N=2]
**CBF/CBV mismatch on CTP** – n(%) [known in]	0 (0) [N=2]	5 (63) [N=8]	2 (40) [N=5]	1 (50) [N=2]

Online Resource 2 – Baseline, procedural and outcome, and imaging variables per EVT and/or IVT treatment. *AChA=anterior choroidal artery, ACOM=anterior communicating artery, CBF=cerebral blood flow, CBV=cerebral blood volume, CTP=CT perfusion, EVT=endovascular treatment, ICA=internal carotid artery, IVT=intravenous thrombolysis, IQR=interquartile range, M1=first segment of middle cerebral artery, min=minutes, mRS=modified Rankin Scale score, n denotes the number of patients, NA=not applicable/available, NIHSS=national institute of health stroke scale, SD=standard deviation, sICH=symptomatic intracranial haemorrhage. If the [known in] number is not shown, the variable was known in all patients.*
