# Supplementary material for: Thrombectomy for acute ischemic stroke patients with isolated distal internal carotid artery occlusion: a retrospective observational study
Source: Neuroradiology. 2020 Oct 7;63(5):777–86. doi: 10.1007/s00234-020-02550-5 (PMC8041676; doi:10.1007/s00234-020-02550-5)
Supplement: Supplementary file 3 — (PDF 152 kb). [file 234_2020_2550_MOESM3_ESM.pdf]

	EVT [N=41]	Non-EVT [N=10]	Registry overall [N=3180]
**No access** – n(%; *95%CI*)	6(15; *7-28*)	NA	185(6; *5-7*) [N=3165]
**Aspiration first** – n(%; *95%CI*) [known in]	5(17; *8-35*) [N=29]	NA	676(21; *20-23*) [N=3165]
**Stent retriever first** – n(%; *95%CI*) [known in]	24(83; *65-92*) [N=29]	NA	1776(56; *54-58)* [N=3165]
**Intracranial atherosclerotic disease present** – n(%; *95%CI*) [known in]	21(53; *38-67*) [N=40]	5(50; *24-76*)	1831(60; *59-62*) [N=3036]
**Emergent carotid stenting** – n(%; *95%CI*) [known in]	4(11; *4-25*) [N=36]	NA	175(6; *5-7*) [N=2989]
**Clot migration** – n(%; *95%CI*)	8(20; *10-34*)	NA	554 (21; *19-22*) [N=2664]

Supplementary Table 2 Procedural data *EVT=endovascular treatment. If the [known in] number is not shown, the variable was known in all patients.*
